# Large extracellular vesicles regulate endothelial angiogenic potential *via* paracrine and autocrine signaling

**DOI:** 10.1016/j.jbc.2026.111193

**Published:** 2026-01-23

**Authors:** Grace Richmond, Rose Nguyen, Alanna Sedgwick, Jeffrey S. Schorey, Crislyn D’Souza-Schorey

**Affiliations:** Department of Biological Sciences, University of Notre Dame, Notre Dame, Indiana, USA

**Keywords:** cancer, endothelial cells, melanoma, extracellular vesicles, tumor microenvironment

## Abstract

Angiogenesis, a process associated with tumor growth and development, is often linked to advanced disease and poor clinical outcomes. Tumor cells establish a proangiogenic microenvironment through the release of paracrine signaling mediators, including extracellular vesicles (EVs). EVs have been shown to facilitate intercellular communication and encompass a diverse range of secreted vesicles, including small EVs, which range in size from ∼60 to 100 nm, and large EVs (L-EVs), which are even more diverse and range from 200 nm to >1 μm in size. Despite advancements in anti-angiogenic cancer therapies, such as bevacizumab, late-stage tumors, including advanced melanomas, exhibit mixed clinical responses. In this study, we elucidate a unique role for melanoma-derived L-EVs in promoting bevacizumab-insensitive endothelial angiogenic phenotypes. L-EV-mediated increase in endothelial tube formation is sensitive to the effects of sorafenib, a multikinase inhibitor, but not SU5416, a selective vascular endothelial growth factor (VEGF)–receptor inhibitor. We also demonstrate that melanoma L-EVs contain VEGF as luminal cargo and induce paracrine effects by modulating the endothelial EV secretome. The release from endothelial cells of soluble VEGFs, EVs, and proangiogenic cytokines, such as interleukin-8, macrophage migration inhibitor factor, and plasminogen activator inhibitor-1, drives sustained endothelial tube formation through autocrine signaling. Finally, we show that EV subtypes have distinct effects on the acquisition of angiogenic phenotypes, and their roles vary with tumor cell type. These findings provide new insight into the mechanisms of angiogenic therapy resistance in melanoma and demonstrate the differential functions of EV subtypes in angiogenesis across tumor types.

Melanoma is the deadliest form of skin cancer, with high rates of metastasis and rising incidence over the last decade. Primary melanoma tumors originate from the transformation of melanocytes in association with UV exposure and grow vertically over time (1, 2, 3). Following vertical growth into dermal skin layers, melanoma tumors invade outward and metastasize with support from blood vessel accumulation into dense vascular networks. Melanoma tumor vessel density has a strong clinical correlation to poor prognosis and outcomes of decreased overall survival and high rates of recurrence (4, 5).

Tumor cells drive angiogenesis through paracrine signaling by releasing signaling molecules, including vascular endothelial growth factor (VEGF), interleukin (IL)-6, and IL-8, that help coordinate the growth and organization of new blood vessels (6, 7). These proangiogenic mediators are released from tumor cells in both soluble form and in extracellular vesicles (EVs). EVs released from tumor cells can interact with nearby cells to deliver protein, lipid, and nucleic acid cargo that may influence the behavior of recipient cells (8, 9, 10). Tumor–EV signaling leads to the conditioning of the tumor microenvironment in part by facilitating cancer-associated fibroblast reprogramming and modulating the activities of immune cells and endothelial cells (ECs) (11, 12, 13, 14). Studies on angiogenesis have implicated EV-associated factors in driving angiogenic phenotypes, but the mechanisms and responsible cargos are not universal across tumor types. Understanding differences in tumor-derived EV contributions is a complex and lingering gap in the field. Most studies have primarily focused on defining the roles of small EVs (sEVs) in angiogenesis. sEVs, including exosomes, are a subtype of EVs, which belong to a larger family of vesicles that also includes large EVs (L-EVs), such as microvesicles (MVs) (15, 16). The roles of the diverse EV subtypes in angiogenesis are relatively underexplored and less understood. Elucidating the roles of EVs in the tumor microenvironment in the modulation of EC behaviors is important to more fully understand the underlying mechanisms and regulation of tumor angiogenesis.

The importance of understanding the role of EVs is underscored by their influence in clinical therapies targeting tumor angiogenesis, particularly those that have had minimal success and often result in drug resistance and tumor recurrence (17, 18). For instance, reports suggest that treatment strategies targeting VEGF with monoclonal antibodies to block angiogenesis are compromised by the EV-mediated transfer of therapy-insensitive VEGF to neighboring cells (19, 20). sEV-associated VEGF isoforms were still able to bind receptors on ECs and stimulate proangiogenic phenotypes in the presence of bevacizumab. Understanding the impacts of EV subtypes on angiogenesis and how they may influence targeted therapies will be highly useful to inform the development of more effective treatments. Here, we describe the role of melanoma-derived L-EVs in the induction of endothelial angiogenic phenotypes. We have compared the effects of sEVs and L-EVs derived from melanoma and other tumor cell lines on ECs. We show that L-EVs promote endothelial tube formation *via* mechanisms distinct from sEVs derived from the same cell lines. Furthermore, we demonstrate that the roles of EV subtypes vary with tumor cell type. Immunoassays and enzymatic digestion experiments reveal that melanoma L-EVs contain VEGF as a luminal cargo and promote endothelial tube formation in a bevacizumab-insensitive manner. In addition, L-EVs promote the release of proangiogenic cytokines and EVs from ECs. Furthermore, cytokine neutralization experiments validate that changes to the endothelial secretome are critical for melanoma L-EV–induced angiogenic potential. These findings collectively show that EV subtypes behave differently in the context of tumor type, and they describe a unique mechanism of bevacizumab resistance in melanoma, thus providing further insight into the mixed clinical success of current therapeutics.

## Results

### Melanoma L-EVs promote endothelial angiogenic phenotypes in a mechanism distinct from sEVs

These investigations were initiated by assessing the effects of the EV subtypes—L-EVs and sEVs, alongside the potent angiogenic factor VEGF, on endothelial tube formation. EVs were isolated as previously described and fractionated into L-EV or sEV populations using differential ultracentrifugation and high-resolution iodixanol (Optiprep) density gradients (Fig. S1, *A*–*D*) (21). Consistent with previous reports, L-EV fractions mainly consisted of MVs based on size and protein composition, including β-actin, ARF6, and Annexin A1, whereas the sEV fractions were smaller in size and enriched in exosome markers, ALIX and CD81 (21).

**Figure 1:**
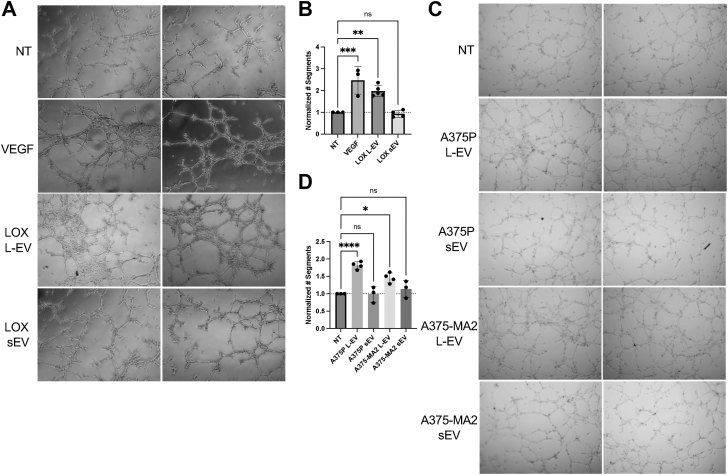
**Melanoma L-EVs increase endothelial tube formation, distinct from sEVs.***A*, SVEC4–10 endothelial cells were incubated with no-treatment 1X PBS control, VEGF (20 ng/ml), or LOX L-EVs or sEVs, as described in the Experimental procedures section. Cells were allowed to form tubes, and networks were imaged on an inverted microscope after 5 h. *B*, resulting tube networks were analyzed for the number of segments using Fiji and normalized to respective no-treatment controls among replicates. *C* and *D*, endothelial cells were incubated with L-EVs or sEVs derived from paired primary and metastatic cell lines A375P or A375-MA2, and the number of segments was quantified using Fiji. Data are presented as means ± SD. The *p* values were obtained by one-way ANOVA with Dunnett’s correction (ns, not significant, ∗*p* < 0.05, ∗∗*p* < 0.01, ∗∗∗*p* < 0.001, and ∗∗∗∗*p* < 0.0001) from at least three independent experiments. L-EV, large extracellular vesicle; sEV, small extracellular vesicle; VEGF, vascular endothelial growth factor.

Endothelial tube formation was examined using a well-established *in vitro* assay for angiogenesis wherein ECs organize to form capillary tube structures (22, 23). We used human umbilical vein endothelial cells (HUVECs) and SVEC4–10 mouse ECs for these studies. To assess the effects of L-EVs, isolated vesicles were incubated with ECs, and resulting capillary networks were analyzed for tube features, including the number of segments, nodes, junctions, and meshes (24). We assessed whether L-EVs were taken up by recipient cells by monitoring in parallel the uptake of fluorescent L-EVs isolated from the melanoma cell line, LOX, expressing GFP-tagged Annexin A1, an MV marker (21, 25). Fluorescent GFP puncta were visible in the recipient cell cytoplasm, indicating that L-EVs were internalized by ECs (Fig. S1*E*). These studies revealed that L-EV uptake promoted robust tube formation in a dose-dependent manner and enhanced elongation of tube networks relative to mock-treated cells and comparable to that observed upon stimulation with VEGF alone (Figs. 1, *A* and *B*, S2*A*, and S1*F*). Notably, however, the melanoma sEV fraction had little to no effect on endothelial tube formation. To confirm that this finding was not exclusively a function of the cell line used, we examined the effect of EVs derived from the paired primary and metastatic melanoma cell lines, A375P and A375-MA2. We observed that L-EVs, but not sEVs, derived from both A375P and A375-MA2 melanoma cell lines increased tube formation (Figs. 1, *C* and *D* and S2*B*). Thus, the effects of EVs on endothelial tube formation appeared to be consistent across melanoma cell lines and independent of tumor stage.

Given prior reports on the effects of sEVs on proangiogenic phenotypes in other tumor cell types (20, 26), we assessed the effects of L-EVs and sEVs on two additional tumor cell lines: MDA-MB-468, a metastatic breast cancer cell line, and 786-O, a clear cell renal carcinoma cell line. MDA-MB-468–derived L-EVs resulted in increased tube formation in similar ways to that described above for melanoma L-EVs (Fig. S3, *A*–*C*), whereas sEVs from the same cell line had no effect on tube formation. The effects of breast cancer cell–derived L-EVs in promoting proangiogenic phenotypes are consistent with prior reports (27). With respect to 786-O cells, however, both the L-EV and sEV fractions increased endothelial tube formation. Furthermore, when compared with the effects of breast and melanoma cell types, the induction of nodes, junctions, and meshes was even stronger with 786-O L-EVs (Fig. S3, *A*–*C*). The sEV fraction from 786-O cells formed dense networks and a distinct sprouting morphology, evident through increased branching. Endothelial branching or sprouting phenotypes are indicative of tip cell formation and are characterized by extensions at the edges of vascular sprouts (28). As such, both the 786-O sEV and L-EV fractions enhanced proangiogenic phenotypes, although in unique ways. These concerted and potent effects of renal EVs may contribute to the highly vascularized nature of clear cell renal cell carcinoma tumors (29). Notably, treatment of ECs with an equivalent amount of EVs from a nontumorigenic fibroblast cell line had no effect on tube formation, indicating that the angiogenic growth–inducing effects are specific to vesicles shed from the tumor cell lines (Fig. S3*D*).

In light of the aforementioned findings, we examined melanoma L-EVs for the presence of proangiogenic cargos identified in EVs shed from various tumor cell types. We first examined for the presence of CD133, which has been shown to be a proangiogenic cargo of L-EVs derived from colorectal cancer cells (30). CD133 was not present in the melanoma cells by Western blotting of cell lysates (Fig. S4, *A* and *B*). We also examined for the presence of EphB2, which was reported to be a proangiogenic cargo on sEVs derived from head and neck squamous cell carcinoma cells (26). We observed that EphB2 was neither expressed in the melanoma and MDA-MB-468 cell lines nor detectable in L-EV and sEV fractions (Fig. S4, *C* and *D*). However, it was expressed in the 786-O clear cell renal cell carcinoma cell line. Furthermore, EphB2 appeared to be enriched in the 786-O sEV fraction (Fig. S4*E*). Taken together, these studies show that the effects of EV subtypes on ECs vary with tumor origin and that key proangiogenic cargos vary between EV subtypes. They also highlight the importance of understanding the distinct roles of EV subtypes in angiogenesis.

### Melanoma L-EVs contain VEGF as a luminal cargo

**Figure 2:**
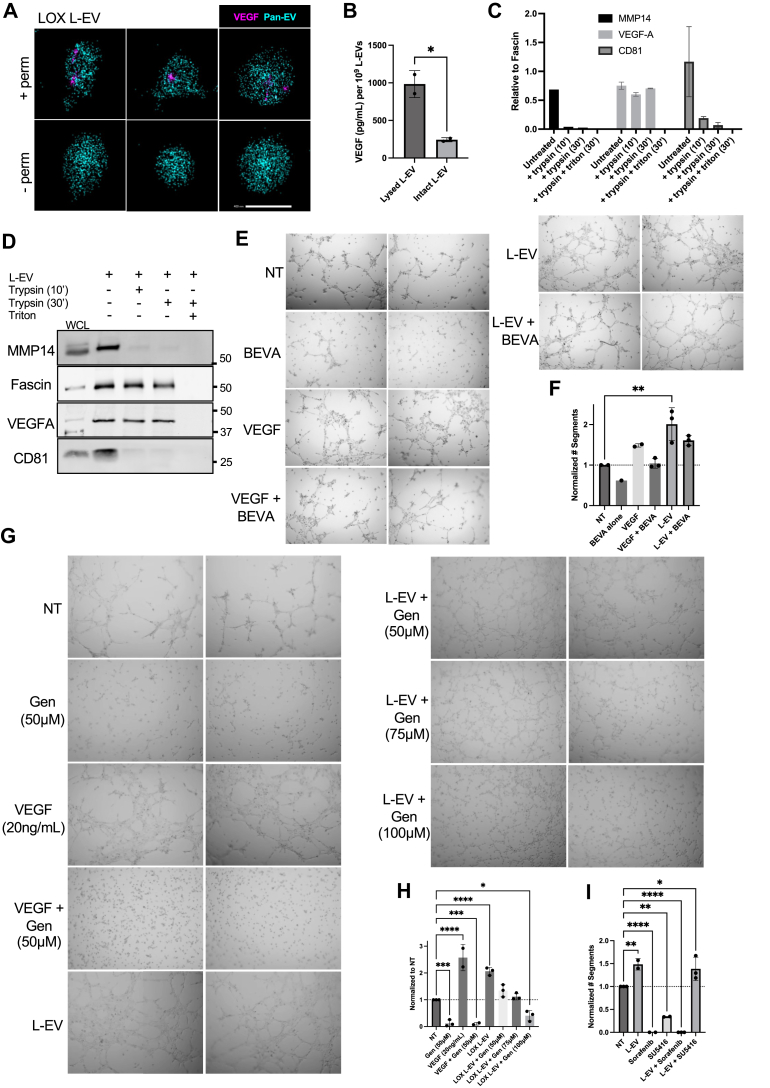
**VEGF-A is primarily a luminal cargo of melanoma L-EVs.***A*, L-EVs were purified from LOX melanoma cells as described in the Experimental procedures section and processed for super-resolution microscopy. EVs were captured using phosphatidylserine and stained with VEGF-A and Pan-EV in the presence (+perm) or absence (-perm) of saponin. The scale bar represents 400 nm. *B*, L-EVs were isolated and resuspended in 0.1 μm filtered 1X PBS or in RIPA lysis buffer. Samples were used to perform a VEGF-A ELISA, and concentrations per 10^9^ L-EVs were calculated based on a four-parameter logistic (4PL) curve fit model generated from standard concentrations. *C* and *D*, the same number of isolated L-EVs (10^9^) was resuspended in 0.1 μm filtered 1X PBS with no treatment, 1× trypsin–EDTA (10 min or 30 min, as indicated), or a combination of 1× trypsin*–*EDTA and 0.1% Triton X-100. L-EVs were reisolated, resuspended in 1.5× loading dye, and separated by SDS-PAGE for Western blot analysis. Pixel densities were quantified using Fiji, and independent replicates were plotted as means ± SD. Molecular weight markers (kilodaltons) are indicated. *E* and *F*, SVEC4–10 endothelial cells were incubated with no-treatment vehicle control (NT), 100 nM bevacizumab (BEVA), 20 ng/ml VEGF, LOX L-EVs, or combination treatments, as described in the Experimental procedures section. Tube networks were imaged after 5 h and analyzed in Fiji for the number of segments. Values among replicates were normalized to respective no-treatment controls. *G*, SVEC4–10 cells were incubated with 50 to 100 μM genistein (Gen), 20 ng/ml VEGF, LOX L-EVs, or combination treatments, as indicated. Tube networks were allowed to form and were imaged after 5 h. *H*, tube analysis was conducted in Fiji to determine the number of segments after normalization to the no-treatment control condition. *I*, SVEC4–10 cells were incubated with 10 μM SU5416 or 20 μM sorafenib alone or in combination with L-EVs. Data are presented as means ± SD. The *p* values were obtained by one-way ANOVA with (*F*) Tukey’s correction or (*H* and *I*) Dunnet’s correction or (*B*) an unpaired two-tailed *t* test (ns, not significant, ∗*p* < 0.05, ∗∗*p* < 0.01, ∗∗∗*p* < 0.001, and ∗∗∗∗*p* < 0.0001) from at least three independent experiments. L-EV, large extracellular vesicle; RIPA, radioimmunoprecipitation assay; VEGF, vascular endothelial growth factor.

Given that VEGF is a major factor that drives angiogenesis, we investigated melanoma L-EVs for VEGF as a cargo using a combination of morphological and biochemical approaches. VEGF is thought to be present on EVs either as luminal or surface membrane–bound cargo (19, 20, 27). At the surface of EVs, VEGF is able to directly bind receptors on recipient cells, whereas luminal VEGF is shielded from direct interactions with recipient cells. We assessed vesicle morphology and localization of VEGF *via* confocal and stochastic optical reconstruction microscopy (STORM) imaging. When L-EVs were processed for confocal imaging in the presence or the absence of a membrane-permeabilizing agent, we observed a 1.5-fold increase in labeling for VEGF-A in the presence of the permeabilizing agent (Fig. S5*A*). L-EVs were marked by labeling with β1 integrin, a transmembrane cargo, that remained constant under both experimental conditions. For super-resolution STORM imaging, melanoma L-EVs were marked with Pan-EV, a membrane dye, with and without permeabilization. VEGF labeling was barely detectable relative to that observed in the presence of the permeabilizing agent (Fig. 2*A*). These studies indicate that the majority of melanoma L-EV–associated VEGF is present in the lumen. Similarly, when L-EV–associated VEGF was examined by ELISA using both intact and lysed L-EVs, the VEGF signal was higher in lysed L-EVs, indicating that the majority of VEGF was present in the L-EV lumen (Fig. 2*B*). Finally, trypsin digestion to degrade surface-bound protein cargo had no effect on L-EV VEGF. As controls, fascin, a known luminal cargo, was not sensitive to trypsin degradation under the same experimental conditions, whereas transmembrane cargos, matrix metalloproteinase 14 (MMP14) and CD81, were sensitive to trypsin. In all cases, L-EV cargos were sensitive to enzymatic digestion in the presence of detergent (Fig. 2, *C* and *D*). Collectively, these results suggest that the majority of VEGF is in the lumen of melanoma L-EVs, with only a relatively small pool of VEGF at the L-EV surface.

### Effect of VEGF neutralization on L-EV–induced phenotypes

Given the aforementioned findings, we investigated the impact of L-EVs in promoting endothelial angiogenic phenotypes in the presence of VEGF-neutralizing antibodies. VEGF-neutralizing antibodies, including bevacizumab, have been used in the clinic as antiangiogenic therapies but with mixed success in melanoma (31). In tube assays, addition of bevacizumab markedly reduced VEGF-induced tube formation to baseline levels (Fig. 2, *E* and *F*). Notably, under the same experimental conditions, melanoma L-EV–treated cells exhibited endothelial tube structures above baseline (Fig. 2, *E* and *F*). This decreased sensitivity to bevacizumab is somewhat analogous to a previous report in breast cancer cell lines, which demonstrated that crosslinked VEGF on MVs interacts with heat shock protein 90 (HSP90) in a manner that renders breast cancer cell lines insensitive to bevacizumab (27). The study also showed that pretreatment with 17-AAG, an HSP90 inhibitor, released VEGF and resensitized the breast MVs to bevacizumab. However, we found that pretreating melanoma L-EVs with 17-AAG had no effect on tube formation (Fig. S5, *B* and *C*). In addition, VEGF interactions with heparin sulfate on the EV surface have also been implicated in bevacizumab insensitivity (20). We ruled out this mechanism, as heparinase treatment had no effect on melanoma L-EV–induced endothelial tube formation (Fig. S5, *D* and *E*). These studies show that VEGF is predominantly a luminal cargo of melanoma L-EVs, which potentially establishes a unique mechanism for bevacizumab insensitivity. Taken together, our findings suggest that there are multiple mechanisms by which EV-associated VEGF may confer bevacizumab insensitivity.

To further investigate the effect of blocking VEGF signaling, ECs were treated with a tyrosine kinase inhibitor, genistein. Genistein is known to inhibit endothelial tube formation (32), and consistently, treatment with 50 μM genistein inhibited tube formation (Fig. 2, *G* and *H*). VEGF-induced tube formation was also blocked under the same experimental conditions. However, the addition of genistein only partially arrested L-EV–induced tube formation. A decrease in tube thickness was observed alongside a dose-dependent decrease in the number of segments but without significant effect on nodes, junctions, tube length, or meshes (Figs. 2, *G* and *H* and S5*F*). At significantly higher concentrations of genistein (100 μM), there was a marked loss of capillary networks, although remnants of tube formation were still observed. These data suggest that L-EVs elicit additional signaling events to drive endothelial tube formation relative to VEGF alone that may involve tyrosine kinase–independent alterations to endothelial tube formation. To investigate this contention further, we examined the effects of SU5416, a selective inhibitor of VEGFR2, and sorafenib, a multikinase inhibitor with potent inhibitory effects on Raf kinases as well as VEGFR2/3, PDGFR-β, FLT3, and c-Kit (33). Treatment with sorafenib alone blocked tube formation, whereas treatment with SU5416 alone resulted in fewer tubes and a partial block in segment formation (Figs. 2*I* and S5*G*). However, the addition of SU5416 had no effect on L-EV–stimulated tube formation, supporting the aforementioned findings that the direct effects of melanoma L-EVs are independent of VEGFR. In contrast, treatment with sorafenib inhibited L-EV–induced tube formation, suggesting that melanoma L-EVs may act through activation of Raf kinases or one of the aforementioned receptors.

### Melanoma L-EVs facilitate autocrine endothelial VEGF signaling

**Figure 3:**
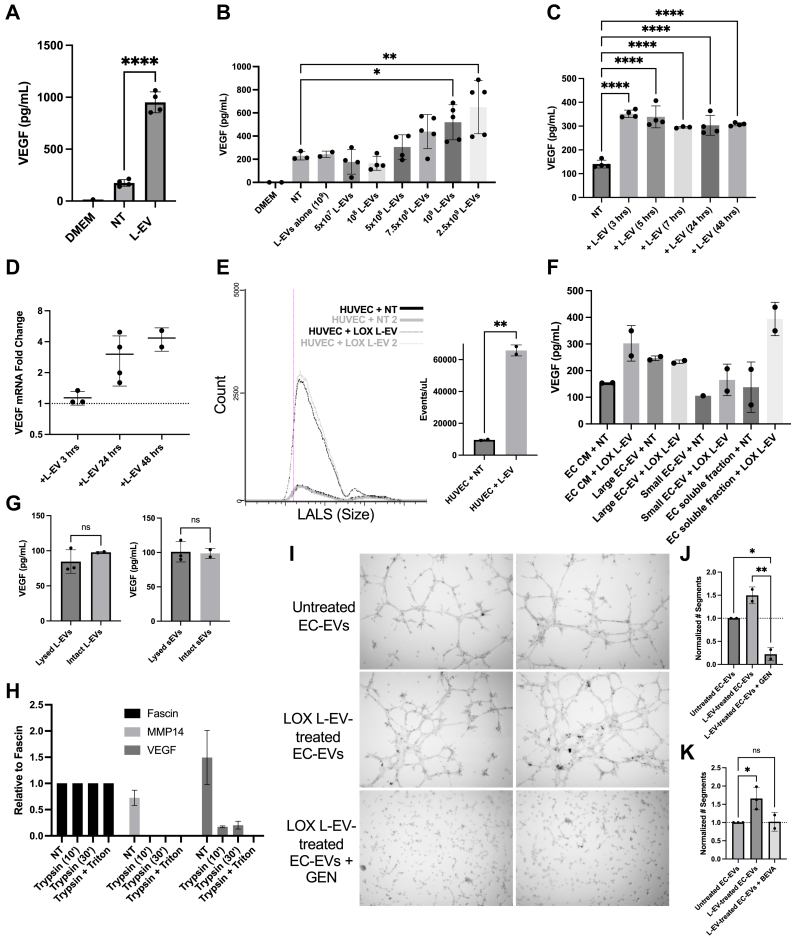
**Melanoma L-EVs alter endothelial VEGF release.***A*, HUVEC cells were treated with 1X PBS control (NT) or LOX L-EVs for 48 h. Conditioned media were collected, and EVs were removed by ultracentrifugation. The remaining supernatant was used for VEGF ELISA analysis. Concentrations were calculated based on a 4PL curve fit model generated from standard concentrations. *B*, HUVEC cells were treated with varying amounts of LOX L-EVs, as indicated. Supernatants were depleted of EVs and used for VEGF ELISA analysis. *C*, HUVEC cells were treated with equivalent amounts of LOX L-EVs for various timepoints, as indicated. Supernatants were depleted of EVs and used for VEGF ELISA analysis. *D*, total RNA was isolated from HUVECs after treatment with equivalent amounts of LOX L-EVs for the indicated times and used to perform qRT–PCR on VEGF-A, as described in the Experimental procedures section. Experiments were repeated at least three times, and outliers were removed. *E*, HUVEC cells were treated with LOX L-EVs or 1X PBS control (NT) for 16 h. Media were removed, and ECs were allowed to shed into EV-depleted media for 72 h. Total EC–EVs were collected and analyzed on a microparticle flow cytometer. Particle count *versus* large-angle light scatter (LALS) was plotted, and particle events per microliter were used to generate a bar graph. *F*, HUVEC cells were treated with LOX L-EVs or 1X PBS (NT) for 16 h, and the media were replaced with EV-free media. Conditioned media (CM) were fractionated and subject to VEGF ELISA analysis. VEGF concentrations were calculated based on a 4PL curve fit model generated from standard concentrations. *G*, L-EVs and sEVs were purified from LOX L-EV–treated or untreated endothelial cells by serial ultracentrifugation, and the resulting EV populations were either lysed in RIPA buffer (“lysed”) or maintained in PBS (“intact”) and subject to a VEGF ELISA. *H*, EC–EVs were collected by ultracentrifugation and used to perform a trypsin digestion as outlined in the Experimental procedures section. EC–EVs were reisolated, resuspended in 1.5× loading dye, and separated by SDS-PAGE for Western blot analysis. Pixel densities were quantified using Fiji and normalized relative to Fascin. *I*–*K*, EC–EVs were collected by ultracentrifugation as outlined above from ECs treated with 1X PBS (untreated) or LOX L-EVs. EC–EVs were used to perform a tube assay in the presence (or absence) of genistein or bevacizumab, as indicated. Adjacent fields of view are shown. Tube networks were analyzed after 5 h in Fiji. Independent biological replicates are plotted as means ± SD. The *p* values were obtained by one-way ANOVA with (*J*) Tukey’s correction or (*A*–*C*, *K*) Dunnett’s correction or (*E* and *G*) an unpaired two-tailed *t* test (ns, not significant, ∗*p* < 0.05, ∗∗*p* < 0.01, ∗∗∗*p* < 0.001, and ∗∗∗∗*p* < 0.0001). EC, endothelial cell; HUVEC, human umbilical vein endothelial cell; L-EV, large extracellular vesicle; 4PL, four-parameter logistic; qRT–PCR, quantitative RT–PCR; RIPA, radioimmunoprecipitation assay; VEGF, vascular endothelial growth factor.

To gain insight into the mechanisms by which melanoma L-EVs promote endothelial capillary tube formation, we first examined the effect of L-EVs on endothelial VEGF production. ECs were incubated with melanoma L-EVs for 48 h, EVs were sedimented by centrifugation, following which conditioned media were examined for soluble VEGF by ELISA. We observed a 5.4-fold increase in soluble VEGF after treatment with melanoma L-EVs (Fig. 3*A*). VEGF release was dose dependent (Fig. 3*B*) and evident as early as 3 h post L-EV treatment (Fig. 3*C*). To determine whether the increased VEGF release involved transcriptional regulation, we examined VEGF mRNA by quantitative RT–PCR (qRT–PCR). VEGF mRNA increased only at the later time points, that is, 24 and 48 h, post L-EV treatment (Fig. 3*D*).

To determine if melanoma L-EV treatment impacts endothelial cell–extracellular vesicle (EC–EV) release, HUVECs were incubated with melanoma L-EVs, followed by microflow cytometry to examine vesicle release. These studies revealed an increase in overall EC–EV shedding (Fig. 3*E*). ELISA analysis of the fractionated conditioned media for VEGF showed that while a significant portion of the L-EV–mediated increase in VEGF secretion is soluble VEGF (Fig. 3*F*), endothelial EVs, both L-EVs and sEVs, also contain VEGF as a surface cargo (Fig. 3*G*). Super-resolution imaging of EC-EVs labeled for VEGF-A with or without permeabilization showed that VEGF is present at the surface of EC–EVs, both large and small (Fig. S6*A*). Likewise, trypsin digestion experiments on the EC–EVs showed that, similar to MMP14, surface VEGF was sensitive to trypsin treatment (Figs. 3*H* and S6*B*).

Next, we assessed tube formation with EVs derived from untreated or LOX L-EV–treated ECs. To this end, EVs released from the same number of ECs were collected. Likely because of increased numbers of EVs, EC–EVs collected from LOX L-EV–treated cells significantly increased all tube features, including the number of segments, meshes, nodes, and junctions relative to EVs released from untreated ECs (Figs. 3, *I* and *J* and S6*C*). Notably, EC–EVs collected from untreated ECs also enhanced segment formation, though to a lesser extent than L-EV–treated EC–EVs (Fig. S6*D*). In addition, genistein treatment of EC–EVs markedly inhibited the effects of EC–EVs (Fig. 3, *I* and *J*). We also tested the effect of bevacizumab with EC–EVs and found that VEGF on the surface of EC–EVs was sensitive to bevacizumab (Figs. 3*K* and S6*E*). Thus, these results suggest that melanoma L-EVs trigger endothelial release of soluble VEGF, as well as EC–EVs with VEGF at the surface, thereby enabling tyrosine kinase–dependent autocrine signaling to drive endothelial tube formation. Taken together, the studies described thus far suggest that melanoma L-EVs contain VEGF as a luminal cargo and induce sustained endothelial tube formation through mechanisms that are both tyrosine kinase dependent and independent. Tyrosine kinase–dependent mechanisms involve altering EC secretion of both soluble VEGF and EC–EV surface–associated VEGF. In addition, the combination treatment of genistein with melanoma L-EVs suggests that mechanisms independent of tyrosine kinase regulation contribute to proangiogenic phenotypes.

### Melanoma L-EVs alter the angiogenic cytokine profile of ECs

**Figure 4:**
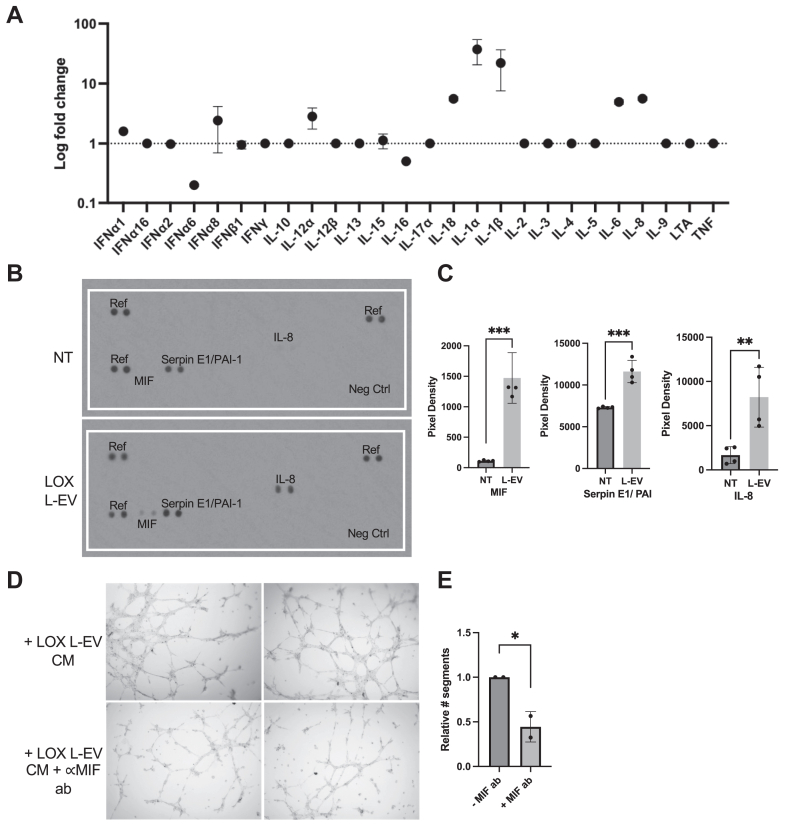
**L-EV-treated ECs exhibit a distinct cytokine profile.***A*, HUVEC cells were treated with LOX L-EVs or 1X PBS control for 48 h, and RNA was collected to perform a qRT–PCR cytokine array. Comparative C_T_ analysis was performed using an 18S endogenous control, and relative expression of each cytokine was plotted. The data are presented as means of two independent replicates. *B*, HUVEC cells were treated as described with either 1X PBS or LOX L-EVs, and conditioned media were collected. EVs were removed, and the remaining culture media were used to perform a cytokine dot blot array. *C*, pixel density analysis of the dot blot array was performed using Fiji, and values were normalized to reference dots. Independent replicates are plotted. *D* and *E*, ECs were treated with LOX L-EVs for 16 h, and EVs were removed. Media were replaced with EV-free, and cells were incubated for 48 h. Conditioned media were used in combination with an MIF-neutralizing antibody (ab) (5 μg/ml) to perform a tube assay. Adjacent fields of view are shown. Data are presented as means ± SD. The *p* values were obtained by an unpaired two-tailed *t* test (ns, not significant, ∗*p* < 0.05, ∗∗*p* < 0.01, ∗∗∗*p* < 0.001, and ∗∗∗∗*p* < 0.0001). EC, endothelial cell; HUVEC, human umbilical vein endothelial cell; L-EV, large extracellular vesicle; MIF, macrophage migration inhibitor factor; qRT–PCR, quantitative RT–PCR.

We investigated other VEGF-independent modes by which L-EVs promote endothelial tube formation. Prior literature demonstrated a role for cytokines in promoting angiogenesis *via* mechanisms that are both VEGF dependent and independent (34). ILs, including IL-1, IL-8, and IL-6, are able to induce expression of VEGF as well as directly induce angiogenic phenotypes, such as proliferation, tube formation, survival, and MMP production (35, 36, 37). We profiled cytokines released by L-EV–treated ECs. To this end, ECs were treated with melanoma L-EVs or mock treated for 48 h, and cellular RNA was collected, followed by cytokine profiling qPCR. Independent biological replicates revealed increased endothelial expression of ILs, IL-12α, IL-18, IL-1 (α/β), IL-6, and IL-8 (Fig. 4*A*) and decreased expression of interferon α6 and IL-16. The alterations in cytokine transcripts were validated by analyzing cytokine and chemokine release from L-EV–treated ECs using a human cytokine antibody array (Fig. 4, *B* and *C*). Independent biological replicates showed increased levels in the culture media of IL-8, macrophage migration inhibitor factor (MIF), and plasminogen activator inhibitor I (Serpin E1), all of which are proangiogenic. Furthermore, to assess the effects of released cytokines on endothelial tube formation, we performed a tube assay with L-EV–depleted EC conditioned media in the presence (or absence) of an MIF-neutralizing antibody. Neutralizing MIF resulted in a reduction in all tube network features (Figs. 4, *D* and *E* and S7) relative to control experimental conditions. This proof-of-principle experiment demonstrates that melanoma L-EVs drive endothelial tube formation in part by regulating endothelial cytokine release.

## Discussion

**Figure 5:**
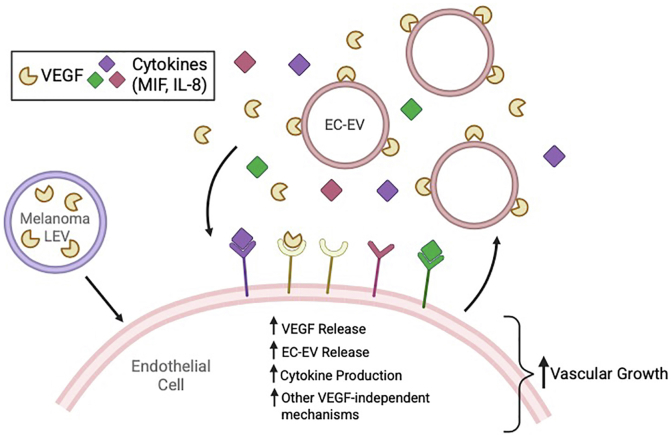
**Schematic depicting the paracrine and autocrine effects of melanoma L-EVs on endothelial angiogenic phenotypes.** Melanoma L-EVs, containing VEGF as a luminal cargo, facilitate paracrine signaling by inducing the secretion of soluble VEGF, EC–EVs, and cytokines from ECs, all of which participate in sustained autocrine signaling to drive vascular growth. EC, endothelial cell; L-EV, large extracellular vesicle; VEGF, vascular endothelial growth factor.

The distinctive mechanisms of cell-to-cell communication offered by sEVS and L-EVs are attracting significant interest in various biological contexts and disease states, including cancer. EVs contribute to cancer progression in various ways and are being explored as targets for new cancer therapies (38, 39). Both L-EVs and sEVs have been suggested to exert major roles in this regard. For instance, EVs have been thought to exert significant influence on tumor angiogenesis, with previous reports showing that one or more nonoverlapping cargos contained in the shed vesicles from various cell types promote proangiogenic phenotypes (40, 41). Here, we report on a unique mechanism by which L-EVs, but not sEVs, shed from melanoma cells induce a robust and sustained response in ECs, triggering endothelial tube formation that is insensitive to the inhibitory actions of bevacizumab, an anti-VEGF therapeutic, as well as SU5416, a VEGF receptor inhibitor. However, the induction of angiogenic phenotypes was sensitive to sorafenib, a multikinase inhibitor. L-EV–induced paracrine stimulation modulates sustained endothelial autocrine behaviors by augmenting the secretion of proangiogenic cytokines and EC-derived EVs (Fig. 5). Finally, we show that the effects of tumor EV subtypes on ECs vary with tumor origin and that key proangiogenic cargos can vary between EV subtypes and across tumor cell lines. These findings are noteworthy in that they elucidate a mechanism by which L-EVs facilitate the induction of proangiogenic phenotypes and provide potential insight into the limited efficacy of bevacizumab in melanoma. Moreover, they also emphasize the importance of understanding the distinct roles of EV subtypes in angiogenesis and highlight the potential of simultaneously targeting the endothelial secretome as a complement to therapeutic strategies for advanced melanoma.

The therapeutic targeting of VEGF to inhibit tumor angiogenesis has yielded mixed outcomes in melanoma. For instance, a retrospective study conducted on advanced melanoma patients indicated that employing bevacizumab as a first- or second-line therapy resulted in limited success, with objective response rates of 6.3% and 3.4%, respectively (42). Other studies have demonstrated limited efficacy when bevacizumab is used alone but enhanced outcomes when combined with interferon or chemotherapy (31, 43). These clinical studies on bevacizumab’s efficacy in melanoma underscore the importance of better understanding the molecular basis of bevacizumab resistance.

Large EVs released by melanoma lines tested here are predominantly MVs based on the particle size of the isolated L-EV fraction; however, we do not exclude the potential contribution of other even L-EV subtypes (44) that may also be present in low abundance. Of note, these proangiogenic EC–EVs by most measures are distinct from the organelle-rich apoptotic body or exopher-like large EC-derived EVs greater than 1 μm that have been shown to promote vasculature damage (45). We demonstrate that melanoma L-EVs harbor VEGF as a luminal cargo, which is shielded from anti-VEGF therapeutic antibodies. Furthermore, inhibition of tyrosine kinase activity resulted in a diminution in endothelial tube formation, indicating that L-EVs induce kinase-dependent, yet VEGF-independent, endothelial activation. L-EV–induced tube formation was also sensitive to sorafenib treatment. Sorafenib, a multikinase inhibitor, has been shown to target ECs by disrupting various proangiogenic pathways, including the receptor tyrosine kinases VEGFR-2/3 and PDGFR-β as well as the Raf–MEK–ERK and PI3K–AKT pathways, with inhibitory effects on cell proliferation, migration, and new vessel formation (33). The drug also affects other pathways, like c-Jun N-terminal kinase, with an impact on cell survival. In future work, it will be of interest to determine which of these pathways are sensitive to sorafenib downstream of L-EVs.

In addition to the studies we describe here, previous reports have also indicated that EVs protect VEGF from therapeutic detection. For example, VEGF isoforms present on the surface of EVs derived from breast tumor and oral squamous carcinoma cell lines, among others, are resistant to bevacizumab treatment, where, depending on the tumor type, interactions between VEGF and EVs mediated by heparan sulfate or HSP90 were reported to be responsible for bevacizumab insensitivity (19, 20, 27). However, here, we found that blocking the effect of heparan sulfate or HSP90 had no impact on melanoma L-EV–induced tube formation. We also note that melanoma L-EV–stimulated ECs released soluble VEGF as well as EVs bearing VEGF on their surface, which were responsive to tyrosine kinase inhibition and bevacizumab treatment. Despite this, the paracrine effects of melanoma L-EVs are insensitive to bevacizumab treatment, pointing to the complexity of bevacizumab sensitivity. Thus, while the luminal positioning of VEGF likely significantly contributes to bevacizumab insensitivity, other mechanisms, including enhanced cytokine release, as discussed below, may also be responsible.

In addition to VEGF secretion, our studies also indicate that L-EVs markedly contribute to the augmentation of soluble cytokine release, namely, MIF, IL-8, and PAI-1 from ECs. MIF is known to facilitate tumor progression and angiogenesis by inducing endothelial proliferation and *in vivo* blood vessel formation (46, 47). IL-8 is another well-established proangiogenic factor that directly influences the proliferation, migration, and tube formation of ECs (35, 48). In fact, in addition to elevated levels of VEGF and other growth factors, IL-8 secretion is also associated with high tumor burden, advanced disease stage, and poor overall survival in melanoma (49). HuMax-IL8, which inhibits IL-8, has been shown to enhance clinical outcomes in gastric and non–small cell lung cancer tumors by reducing serum IL-8 levels and sensitizing tumors to other immunotherapies, including PD-1/PD-L1, although there are no reports on IL-8 inhibition on clinical outcomes in melanoma (50, 51). The urokinase-type plasminogen activator receptor–system is also recognized as a regulator of angiogenesis, activating MMPs to degrade extracellular matrix and release matrix-bound factors, such as VEGF (52). Thus, L-EVs may also indirectly promote angiogenesis by altering the urokinase-type plasminogen activator receptor system. We note that the expression of several cytokines, including IL-12α, IL-18, IL-1 (α/β), and IL-6, was increased in response to melanoma L-EVs. It is possible that some of these cytokines may also be EV associated although this remains to be ascertained. Notably in this regard, serum cytokine levels in melanoma are often correlated with poor prognosis and late disease stage (53). Increased IL-6 is a prognostic factor for shorter overall survival in advanced melanoma patients receiving immune checkpoint inhibitors or chemotherapy (54).

There is much that remains to be understood regarding the mechanisms by which melanoma L-EVs contribute to tumor angiogenesis. Areas of future research include identifying the surface molecules essential for L-EV–EC interactions and cargos responsible for promoting angiogenic phenotypes. In addition, the activation of VEGF-dependent and -independent signaling pathways in ECs in response to L-EVs warrants further investigation. Finally, investigating the efficacy of sorafenib therapies or the synergistic effects of blocking cytokines with antiangiogenic therapy in models of metastatic melanoma, where bevacizumab efficacy demonstrates variability, could provide valuable insights. Although the answers to these and other questions surrounding L-EV function remain elusive, it is evident that the actions of L-EVs and other EVs in tumor progression hold significant promise as exciting research areas with the potential to profoundly impact future translational efforts.

## Experimental procedures

### Cell lines

LOX melanoma cells were kindly provided by Prof Oystein Fodstad, Oslo University, Norway. A375P (Research Resource Identifier [RRID]: CVCL_6233), A375-MA2 (RRID: CVCL_X495), MDA-MB-468 (RRID: CVCL_0419), 786-O (RRID: CVCL_1051), SVEC4–10 (RRID: CVCL_4393), HUVEC (RRID: CVCL_9Q53), and BJ Fibroblast (RRID: CVCL_3653) cell lines were purchased from American Type Culture Collection. LOX were cultured in RPMI supplemented with 10% Atlas serum, 2 mM l-glutamine, and 100 U/ml penicillin–streptomycin; 786-O were cultured in the same way as LOX cells, with the addition of 1 mM sodium pyruvate. A375P, A375-MA2, MDA-MB-468, SVEC4–10, and BJ fibroblast cells were cultured in Dulbecco's modified Eagle's medium supplemented with 10% Atlas serum, 2 mM l-glutamine, 1 mM sodium pyruvate, 100 U/ml penicillin–streptomycin, and SVEC4–10 with heat-inactivated serum. HUVECs were cultured per the manufacturer’s instructions in endothelial growth media (Lonza, catalog no.: CC-3162). All cell lines were routinely checked to ensure the absence of mycoplasma contamination and maintained at 37 °C in a humidified chamber containing 5% CO_2._

### Antibodies and other reagents

Antibodies to β1 integrin (AIIB2) and CD133 (0.5 μg/ml) were purchased from the Developmental Studies Hybridoma Bank at the University of Iowa; antibodies to ALIX, β-actin, VEGF-A, CD81, and ARF6 were purchased from Protein Tech; and antibodies to β1 integrin (1952), MMP14, and Fascin were purchased from Millipore. EphB2 antibody was purchased from Cell Signaling. The Pan-EV stain used for STORM imaging was from ONI. Secondary Alexa Fluor Plus antibodies and phalloidin for immunofluorescence and Western blotting were purchased from ThermoFisher. All antibodies were purchased from commercial sources and have been extensively validated by the manufacturer. Western blot analysis confirmed antibody specificity, showing bands at expected molecular weights. Relevant positive and negative control lysates were used.

### EV isolation

Cells were expanded to reach ∼80% confluency when growth media were replaced with media supplemented with EV-depleted serum (Gibco, ThermoFisher) for up to 3 days, and media were collected. EVs were isolated by serial ultracentrifugation and extensively washed with PBS and/or further purified using an iodixanol density gradient, as previously described (21, 25). All EV fractions were analyzed for particle concentration and size using a micro flow cytometer (Apogee Flow Systems). Calibration gates were determined using a mixture of silica beads at 180, 240, 300, 590, 880, and 1300 nm diameters. Data analysis was done using the Histogram Software (Apogee Flow Systems).

### Endothelial tube formation assay

For the endothelial tube formation assay, SVEC4–10 ECs (3 × 10^4^) were plated onto low growth factor Matrigel (Corning) in serum-free Dulbecco's modified Eagle's medium with 2% heat-inactivated FBS plus either EVs (5 × 10^6^–1 × 10^9^), VEGF-A (20 ng/ml), or genistein (50–100 μM). Endothelial tube formation was monitored at 37 °C and imaged on a Zeiss Axio Observer. Analysis of tube networks was conducted using the Angiogenesis Analyzer ImageJ plugin (24). For SU5416 and sorafenib experiments, L-EVs were added to the tube assays in combination with the indicated drugs at 10 μM and 20 μM concentrations, respectively. Where indicated, L-EVs were pretreated with 100 nM bevacizumab (MCE) or 0.9 mU/ml *Bacteroides* Heparinase I (New England Biolabs) at 37 °C. L-EVs were reisolated and added to tube assays described above. For assays involving MIF neutralization, ECs were incubated with isolated L-EVs, media were replaced, and conditioned media were collected after 48 h and used in combination with MIF-neutralizing antibody (5 μg/ml; R&D Systems) in tube assays. For 17-AAG treatments, LOX cells were treated with 17-AAG inhibitor (500 nM–1 μM; MCE) or an equivalent amount of dimethyl sulfoxide in EV-free media, and cells were allowed to shed for 24 h before L-EV isolation. For combined 17-AAG and bevacizumab treatment, L-EVs were pretreated with 17-AAG (10 μM) for 90 min at 37˚C and then used in combination with bevacizumab (0.5 μg/ml) in tube assays. All treatment conditions were normalized to a no-treatment vehicle control independently for each experiment.

### Western blotting

Cells were lysed in radioimmunoprecipitation assay buffer containing 25 mM Tris–HCl (pH 7.6), 150 mM NaCl, 1% sodium deoxycholate, 0.1% SDS, and 1% Triton X-100. Immediately prior to use, 1× phosphatase inhibitor and mammalian protease inhibitor cocktail (MilliporeSigma) were added. Lysates were processed for protein separation by SDS-PAGE and transferred to an Immobilon-FL polyvinylidene fluoride membrane (MilliporeSigma). Membrane blocking was performed in either 5% nonfat milk or 5% bovine serum albumin (BSA; MilliporeSigma) in Tris-buffered saline (TBS) for 1 h at room temperature. Primary antibodies (diluted per the manufacturer’s instructions) were incubated in 2% nonfat milk or BSA in TBS + 0.2% Tween-20 overnight at 4 °C. Membranes were washed in TBS + 0.1% Tween-20, incubated with Alexa Fluor Plus secondary antibodies diluted 1:10,000 in 2% nonfat milk or BSA in TBS + 0.2% Tween-20 for 1 h at room temperature in the dark. Membranes were again washed in TBS + 0.1% Tween-20 and then in TBS alone. A LiCor Odyssey Scanner was used to image the blots, and pixel densitometry was performed in Fiji (Open Source License). COLO205 lysates were provided by Cell Signaling as a positive control.

### Trypsin digestions

EVs were incubated in 100 μl of 0.1 μm 1× PBS with 1× trypsin–EDTA or 0.1% Triton X-100 (final concentrations) for 10 to 30 min at 37˚C. Enzymes and detergents were diluted by the addition of 900 μl of 0.1 μm 1× PBS. EVs were reisolated by centrifugation.

### Immunofluorescence

Cells were plated on glass coverslips, or EVs were plated on poly-l-lysine (MilliporeSigma)–coated coverslips and allowed to adhere at 37 °C before fixing in 2% paraformaldehyde (Electron Microscopy Supply). Coverslips were washed three times with PBS plus glycine and blocked/permeabilized in 5% BSA with 0.2% Triton X-100 and 0.05% Tween-20. For experiments without permeabilization, the blocking solution only contained 5% BSA. Coverslips were incubated in primary antibodies and further processed for immunofluorescence microscopy as previously described (21). To monitor L-EV uptake, cells were incubated with labeled L-EVs (isolated from the growth media of cells expressing GFP-tagged Annexin A1). Coverslips were imaged on a Leica Stellaris 8 DIVE confocal microscope using the 60× and 100× objectives. For super-resolution STORM microscopy, EVs were captured on prepared assay chips (EV Profiler Kit; Oxford Nanoimaging) and stained with VEGF-A and Pan-EV in the presence or the absence of permeabilizing agent (saponin). Samples were imaged with the ONI Nanoimager (Oxford Nanoimaging). For EC–EVs, particle sizes were analyzed, and EC–EVs sized <200 nm were binned as sEVs and >200 nm were binned as L-EVs.

### Cytokine array

Conditioned media from HUVEC cultures were harvested, spun at 100,000*g*, and the supernatants were tested for cytokine–chemokine levels using the Proteome Profiler Human Cytokine Array Kit (R&D Systems) according to the manufacturer’s instructions. Briefly, supernatants were mixed with a cocktail of biotinylated detection antibodies and incubated on nitrocellulose membranes that were precoated with 36 capture antibodies in duplicates. Cytokine–antibody complexes were immobilized by the capture antibody on the membrane, and unbound material was washed off. Membranes were incubated with streptavidin–horseradish peroxidase, followed by chemiluminescent detection (ThermoFisher). Membranes were exposed to X-ray film, and dot intensities were measured using Fiji.

### VEGF ELISA

For ELISA analysis, conditioned media from HUVEC cultures were tested for VEGF-A by ELISA (ThermoFisher) per the manufacturer’s instructions. Cell culture media were also tested alone to account for background levels of VEGF.

### Quantitative PCR

RNA was isolated from HUVEC cultures, followed by RT using the TaqMan Fast Advanced Cells-to-C_T_ kit (ThermoFisher) per the manufacturer’s instructions. qPCR was performed on a QuantStudio 5 real-time PCR machine (Applied Biosystems) and analyzed by the comparative C_T_ method. Amplification was measured using TaqMan gene expression assays for VEGF-A and 18S (ThermoFisher). Cycling conditions were set according to the manufacturer’s instructions. In separate experiments, complementary DNA was generated from HUVECs using the TaqMan Fast Advanced Cells-to-C_T_ kit (Invitrogen). qPCR was performed on a Step-One-Plus real-time PCR machine (Applied Biosystems) using a TaqMan Array Human Cytokine Network plate (Applied Biosystems) per the manufacturer’s instructions. Briefly, complementary DNA was mixed with TaqMan Fast Advanced Master Mix and added to a plate that was precoated with 28 cytokine and 4 endogenous control assays. Reactions were cycled according to the manufacturer’s instructions and analyzed by the comparative C_T_ method.

### Statistical analysis

Statistical analysis was performed using GraphPad Prism 10, version 10.4.2 (GraphPad Soiftware Inc), or Microsoft Excel (Microsoft Corporation), version 16.97.2. Student’s *t* test was used to compare two groups with data that appeared to be normally distributed with similar variances. When comparing multiple treatment groups to a single control and the data were normally distributed, we performed one-way ANOVA with Dunnett’s correction for multiple comparisons. When multiple groups were analyzed, and each group was compared with all other groups, and the data appeared normally distributed, we used one-way ANOVA with Tukey's test to correct for multiple comparisons. Experiments were performed with at least three biological replicates under similar conditions. For the analysis of the tube formation assay, treatment conditions were normalized to the no-treatment vehicle control for each assay independently. For imaging experiments, representative images are shown with conclusions drawn from at least 10 fields of view across replicates. Additional statistical details are provided in figure legends.

## Data availability

The data supporting the findings of this study are available within the article and its supporting information. Requests for additional information may be directed to the corresponding author.

## Supporting information

This article contains supporting information.

## Conflict of interest

The authors declare that they have no conflicts of interest with the contents of this article.

## References

[1] Waseh S., Lee J.B. (2023). Advances in melanoma: epidemiology, diagnosis, and prognosis. Front. Med. (Lausanne).

[2] Arnold M., Singh D., Laversanne M., Vignat J., Vaccarella S., Meheus F. (2022). Global Burden of Cutaneous Melanoma in 2020 and Projections to 2040. JAMA Dermatol..

[3] Arnold M., de Vries E., Whiteman D.C., Jemal A., Bray F., Parkin D.M. (2018). Global burden of cutaneous melanoma attributable to ultraviolet radiation in 2012. Int. J. Cancer.

[4] Kashani-Sabet M., Sagebiel R.W., Ferreira C.M.M., Nosrati M., Miller J.R. (2002). Tumor vascularity in the prognostic assessment of primary cutaneous melanoma. J. Clin. Oncol..

[5] Ribatti D., Annese T., Longo V. (2010). Angiogenesis and melanoma. Cancers (Basel).

[6] Liu Z.L., Chen H.H., Zheng L.L., Sun L.P., Shi L. (2023). Angiogenic signaling pathways and anti-angiogenic therapy for cancer. Signal. Transduct. Target. Ther..

[7] Lázár-Molnár E., Hegyesi H., Tóth S., Falus A. (2000). Autocrine and paracrine regulation by cytokines and growth factors in melanoma. Cytokine.

[8] Latifkar A., Hur Y.H., Sanchez J.C., Cerione R.A., Antonyak M.A. (2019). New insights into extracellular vesicle biogenesis and function. J. Cell Sci..

[9] O’Brien K., Breyne K., Ughetto S., Laurent L.C., Breakefield X.O. (2020). RNA delivery by extracellular vesicles in mammalian cells and its applications. Nat. Rev. Mol. Cell Biol..

[10] Minciacchi V.R., Freeman M.R., Di Vizio D. (2015). Extracellular vesicles in cancer: exosomes, microvesicles and the emerging role of large oncosomes. Semin. Cell Dev. Biol..

[11] Ko S.Y., Naora H. (2020). Extracellular vesicle membrane-associated proteins: emerging roles in tumor angiogenesis and anti-angiogenesis therapy resistance. Int. J. Mol. Sci..

[12] Zhang D.X., Vu L.T., Ismail N.N., Le M.T.N., Grimson A. (2021). Landscape of extracellular vesicles in the tumour microenvironment: interactions with stromal cells and with non-cell components, and impacts on metabolic reprogramming, horizontal transfer of neoplastic traits, and the emergence of therapeutic resistance. Semin. Cancer Biol..

[13] Sheehan C., D’Souza-Schorey C. (2019). Tumor-derived extracellular vesicles: molecular parcels that enable regulation of the immune response in cancer. J. Cell Sci..

[14] Peng Z., Tong Z., Ren Z., Ye M., Hu K. (2023). Cancer-associated fibroblasts and its derived exosomes: a new perspective for reshaping the tumor microenvironment. Mol. Med..

[15] Jeppesen D.K., Zhang Q., Franklin J.L., Coffey R.J. (2023). Extracellular vesicles and nanoparticles: emerging complexities. Trends Cell Biol..

[16] Clancy J.W., Boomgarden A.C., D’Souza-Schorey C. (2021). Profiling and promise of supermeres. Nat. Cell Biol..

[17] Bergers G., Hanahan D. (2008). Modes of resistance to anti-angiogenic therapy. Nat. Rev. Cancer.

[18] Ye Z.W., Yu Z.L., Chen G., Jia J. (2023). Extracellular vesicles in tumor angiogenesis and resistance to anti-angiogenic therapy. Cancer Sci..

[19] Zhou J., Liu X., Dong Q., Li J., Niu W., Liu T. (2024). Extracellular vesicle-bound VEGF in oral squamous cell carcinoma and its role in resistance to Bevacizumab Therapy. Cancer Cell Int.

[20] Ko S.Y., Lee W.J., Kenny H.A., Dang L.H., Ellis L.M., Jonasch E. (2019). Cancer-derived small extracellular vesicles promote angiogenesis by heparin-bound, bevacizumab-insensitive VEGF, independent of vesicle uptake. Commun. Biol..

[21] Clancy J.W., Sheehan C.S., Boomgarden A.C., D’Souza-Schorey C. (2022). Recruitment of DNA to tumor-derived microvesicles. Cell Rep..

[22] Montesano R., Orci L., Vassalli P. (1983). *In vitro* rapid organization of endothelial cells into capillary-like networks is promoted by collagen matrices. J. Cell Biol..

[23] Goodwin A.M. (2007). In vitro assays of angiogenesis for assessment of angiogenic and anti-angiogenic agents. Microvasc. Res..

[24] Carpentier G., Berndt S., Ferratge S., Rasband W., Cuendet M., Uzan G. (2020). Angiogenesis analyzer for ImageJ — a comparative morphometric analysis of “Endothelial Tube Formation Assay” and “Fibrin Bead Assay”. Sci. Rep..

[25] Jeppesen D.K., Fenix A.M., Franklin J.L., Higginbotham J.N., Zhang Q., Zimmerman L.J. (2019). Reassessment of exosome composition. Cell.

[26] Sato S., Vasaikar S., Eskaros A., Kim Y., Lewis J.S., Zhang B. (2019). EPHB2 carried on small extracellular vesicles induces tumor angiogenesis via activation of ephrin reverse signaling. JCI Insight.

[27] Feng Q., Zhang C., Lum D., Druso J.E., Blank B., Wilson K.F. (2017). A class of extracellular vesicles from breast cancer cells activates VEGF receptors and tumour angiogenesis. Nat. Commun..

[28] Gerhardt H., Golding M., Fruttiger M., Ruhrberg C., Lundkvist A., Abramsson A. (2003). VEGF guides angiogenic sprouting utilizing endothelial tip cell filopodia. J. Cell Biol..

[29] Qian C.N., Huang D., Wondergem B., Teh B.T. (2009). Complexity of tumor vasculature in clear cell renal cell carcinoma. Cancer.

[30] Kim B., Kim S., Park S., Ko J. (2024). CD133-containing microvesicles promote colorectal cancer progression by inducing tumor angiogenesis. Heliyon.

[31] Varker K.A., Biber J.E., Kefauver C., Jensen R., Lehman A., Young D. (2007). A randomized phase 2 trial of bevacizumab with or without daily low-dose interferon alfa-2b in metastatic malignant melanoma. Ann. Surg. Oncol..

[32] Sharifi-Rad J., Quispe C., Imran M., Rauf A., Nadeem M., Gondal T.A. (2021). Genistein: an integrative overview of its mode of action, pharmacological properties, and health benefits. Oxid. Med. Cell Longev..

[33] Wilhelm S.M., Adnane L., Newell P., Villanueva A., Llovet J.M., Lynch M. (2008). Preclinical overview of sorafenib, a multikinase inhibitor that targets both Raf and VEGF and PDGF receptor tyrosine kinase signaling. Mol. Cancer Ther..

[34] Geindreau M., Bruchard M., Vegran F. (2022). Role of cytokines and chemokines in angiogenesis in a tumor context. Cancers (Basel).

[35] Li A., Dubey S., Varney M.L., Dave B.J., Singh R.K. (2003). IL-8 directly enhanced endothelial cell survival, proliferation, and matrix metalloproteinases production and regulated angiogenesis. J. Immunol..

[36] Wei L.-H., Kuo M.-L., Chen C.-A., Chou C.-H., Lai K.-B., Lee C.-N. (2003). Interleukin-6 promotes cervical tumor growth by VEGF-dependent angiogenesis via a STAT3 pathway. Oncogene.

[37] Strozyk E.A., Desch A., Poeppelmann B., Magnolo N., Wegener J., Huck V. (2014). Melanoma-derived IL-1 converts vascular endothelium to a proinflammatory and procoagulatory phenotype via NFκB activation. Exp. Dermatol..

[38] Clancy J.W., D’souza-Schorey C. (2023). Tumor-Derived extracellular vesicles: multifunctional entities in the tumor microenvironment. Annu. Rev. Pathol. Mech. Dis..

[39] Chang W.H., Cerione R.A., Antonyak M.A. (2021). Extracellular vesicles and their roles in cancer progression. Methods Mol. Biol..

[40] Huang M., Lei Y., Zhong Y., Chung C., Wang M., Hu M. (2021). New insights into the regulatory roles of extracellular vesicles in tumor angiogenesis and their clinical implications. Front. Cell Dev. Biol..

[41] Todorova D., Simoncini S., Lacroix R., Sabatier F., Dignat-George F. (2017). Extracellular vesicles in angiogenesis. Circ. Res..

[42] Cui C., Yan X., Liu S., Deitz A.C., Si L., Chi Z. (2019). Real-world clinical outcomes of anticancer treatments in patients with advanced melanoma in China: retrospective, observational study. Int. J. Surg. Oncol. (N Y).

[43] Han X., Ge P., Liu S., Yang D., Zhang J., Wang X. (2023). Efficacy and safety of bevacizumab in patients with malignant melanoma: a systematic review and PRISMA-compliant meta-analysis of randomized controlled trials and non-comparative clinical studies. Front. Pharmacol..

[44] D’Souza-Schorey C., Di Vizio D. (2025). A class of large cell-like extracellular vesicles: extracellular vesicles. Nat. Cell Biol..

[45] Atkin-Smith G.K., Santavanond J.P., Light A., Rimes J.S., Samson A.L., Er J. (2024). In situ visualization of endothelial cell-derived extracellular vesicle formation in steady state and malignant conditions. Nat. Commun..

[46] Chesney J., Metz C., Bacher M., Peng T., Meinhardt A., Bucalal R. (1999). An essential role for macrophage Migration Inhibitory Factor (MIF) in angiogenesis and the growth of a murine lymphoma the role of MIF in. Mol. Med..

[47] Mitchell R.A., Bucala R. (2000). Tumor growth-promoting properties of macrophage migration inhibitory factor (MIF). Semin. Cancer Biol..

[48] Shi J., Wei P.K. (2016). Interleukin-8: a potent promoter of angiogenesis in gastric cancer. Oncol. Lett..

[49] Ugurel S., Rappl G., Tilgen W., Reinhold U. (2001). Increased serum concentration of angiogenic factors in malignant melanoma patients correlates with tumor progression and survival. J. Clin. Oncol..

[50] Kargl J., Zhu X., Zhang H., Yang G.H.Y., Friesen T.J., Shipley M. (2019). Neutrophil content predicts lymphocyte depletion and anti-PD1 treatment failure in NSCLC. JCI Insight.

[51] Bilusic M., Heery C.R., Collins J.M., Donahue R.N., Palena C., Madan R.A. (2019). Phase i trial of HuMax-IL8 (BMS-986253), an anti-IL-8 monoclonal antibody, in patients with metastatic or unresectable solid tumors. J. Immunother. Cancer.

[52] Ismail A.A., Shaker B.T., Bajou K. (2022). The plasminogen–activator plasmin system in physiological and pathophysiological angiogenesis. Int. J. Mol. Sci..

[53] Wang X., Montoyo-Pujol Y.G., Bermudez S., Corpas G., Martin A., Almazan F. (2021). Serum cytokine profiles of melanoma patients and their Association with tumor progression and metastasis. J. Oncol..

[54] Laino A.S., Woods D., Vassallo M., Qian X., Tang H., Wind-Rotolo M. (2020). Serum interleukin-6 and C-reactive protein are associated with survival in melanoma patients receiving immune checkpoint inhibition. J. Immunother. Cancer.

