# Supplementary material for: Large extracellular vesicles regulate endothelial angiogenic potential via paracrine and autocrine signaling
Source: J Biol Chem. 2026 Jan 23;302(3):111193. doi: 10.1016/j.jbc.2026.111193 (PMC12907857; doi:10.1016/j.jbc.2026.111193)
Supplement: Supporting information [file mmc1.pdf]

Supporting Information

Large extracellular vesicles regulate endothelial angiogenic potential via paracrine and autocrine signaling

Grace Richmond, Rose Nguyen, Alanna Sedgwick, Jeff Schorey, and Crislyn D'Souza-Schorey
Department of Biological Sciences, University of Notre Dame.

Supplemental Figure 1. (A) L-EVs and sEVs were isolated from the conditioned media of LOX melanoma cells by serial ultracentrifugation and fractions were run on a micro particle flow cytometer. Histogram plots were generated as large angle light scatter (LALS) vs particle count and representative plots are shown. (B) Equal amounts of EV and whole cell lysates (WCL) were separated by SDS-PAGE and cargo contents were analyzed by western blotting. Molecular weight markers (kD) are indicated. (C,D) Isolated L-EV (C) or sEV (D) populations were purified on an iodixanol density gradient, as described in the methods, and contents were analyzed by western blotting for EV markers. (E) ANXA1-GFP tagged LOX L-EVs were incubated with HUVEC cells for 48 hours and cells were fixed and stained with phalloidin. Confocal microscopy reveals uptake of GFP-L-EVs by ECs with white arrows demonstrating internalized vesicles. (F) Extension of experiment in Figure 1A. Isolated L-EV and sEV populations were used in a tube formation assay. The no-treatment experimental control was the same as in Figure 1A. The number of branches was quantified and normalized to the no treatment control. Values were plotted as means ± SD and the p-values were obtained by one-way ANOVA with Dunnett's correction. (ns: not significant, *$p<0.05$, ** $p<0.01$, ***$p<0.001$, ****$p<0.0001$).

Supplemental Figure 2. (A,B) Full network analyses for tube assays treated with melanoma L-EVs and sEVs. Data are presented as means ± SD. $n \geq 3$.

Supplemental Figure 3. (A-C) L-EVs and sEVs were isolated from MDA-MB-468 and 786-O tumor cells, as described in the methods, and used to perform a tube assay. Networks were imaged after 5 hours and analyzed in Fiji with normalization to the no treatment control. Adjacent fields of view are shown. The p-values were obtained by one-way ANOVA with Tukey's correction. (D) EVs were isolated from non-tumorigenic BJ fibroblast cells and incubated with SVEC4-10 endothelial cells in a tube assay. Resulting networks were imaged after 5 hours, and number of segments was plotted normalized to the no treatment control as means ± SD. The p-value was obtained by an unpaired two-tailed t test. (ns: not significant, *$p<0.05$, ** $p<0.01$, ***$p<0.001$, ****$p<0.0001$). Bar graphs were constructed from at least 3 independent experiments.

Supplemental Figure 4. Corresponding whole cells (WCL), L-EVs and sEVs for each tumor cell line were lysed and separated by SDS-PAGE. The cargo contents were analyzed by western blotting. CD133 (A,B) and EphB2 (C-E) levels were compared to positive control lysates and β actin or β1 integrin were used as loading controls. Pixel densities were measured in Fiji. Data are presented as means ± SD. Molecular weight markers (kD) are indicated.

Supplemental Figure 5. (A) L-EVs were purified from LOX melanoma cells as described in the methods and allowed to adhere on poly-L-lysine-coated coverslips. Vesicles were fixed and

stained with VEGF-A and β1-integrin in the presence (+ perm) or absence (-perm) of Triton-X-100 and Tween-20. Scale bar, 0.5μm. Percent of vesicles that co-stained with VEGF-A under each condition was manually quantified and plotted as means ± SD. The experiment was repeated with quantitation of at least 22 fields per coverslip. The p-value was obtained by an unpaired two-tailed t test. (B) LOX cells were treated with 17-AAG (1μM) or DMSO control and allowed to shed for 24 hours. L-EVs were collected from the conditioned media by serial ultracentrifugation and used to perform a tube formation assay. Number of segments was quantified after 5 hours using Fiji and normalized to the no treatment condition. (C) L-EVs were pre-treated with 17-AAG (10μM) for 90 minutes at 37˚C and then used in combination with bevacizumab (BEVA) (0.5μg/mL) for a tube assay. Number of segments was quantified using Fiji and normalized to the no treatment condition. (D,E) LOX L-EVs were pre-treated with heparinase (0.9mU/mL) for 16 hours prior to use with a tube formation assay. Number of segments was quantified after 5 hours using Fiji. The p-values were obtained by one-way ANOVA with Tukey's correction. (F) Full tube network analysis for genistein (Gen) tube assay experiments. (G) SVEC4-10 cells were incubated with 10μM SU5416 or 20μM Sorafenib alone or in combination with L-EVs, cells allowed to form tubes and imaged after 5 hours. Independent biological replicates are presented as means ± SD. (ns: not significant, *p<0.05, ** p<0.01, ***p<0.001, ****p<0.0001).

Supplemental Figure 6. (A) EC-EVs were collected from LOX L-EV-treated HUVEC cells as described in the methods and processed for super resolution microscopy. Vesicles were fixed and stained with VEGF-A and Pan-EV in the presence (+ perm) or absence (-perm) of saponin. EC-EVs were binned as large (>200nm) or small (<200nm). Scale bars, 500nm (left) and 400nm

(right). (B) EC-EVs were collected by ultracentrifugation and used to perform a trypsin digestion, as outlined in the methods. EC-EVs were re-isolated, re-suspended in 1.5X loading dye, and separated by SDS-PAGE for western blot analysis. Molecular weight markers (kD) are indicated. (C) Full tube network analysis for EC-EV experiments. (D) Extension of experiment in 3I. EC-EVs were collected from untreated HUVEC cells and used in a tube formation assay. The no-treatment control was the same as in Figure 3I. Networks were imaged after 5 hours and number of segments was quantified and plotted as means ± SD after normalization. (E) EC-EVs were used alone or in combination with 100nM bevacizumab for a tube formation assay. Networks were imaged after 5 hours.

Supplemental Figure 7. (A) Full tube network analysis for MIF neutralization experiments. Independent replicates are plotted as means ± SD after normalization.

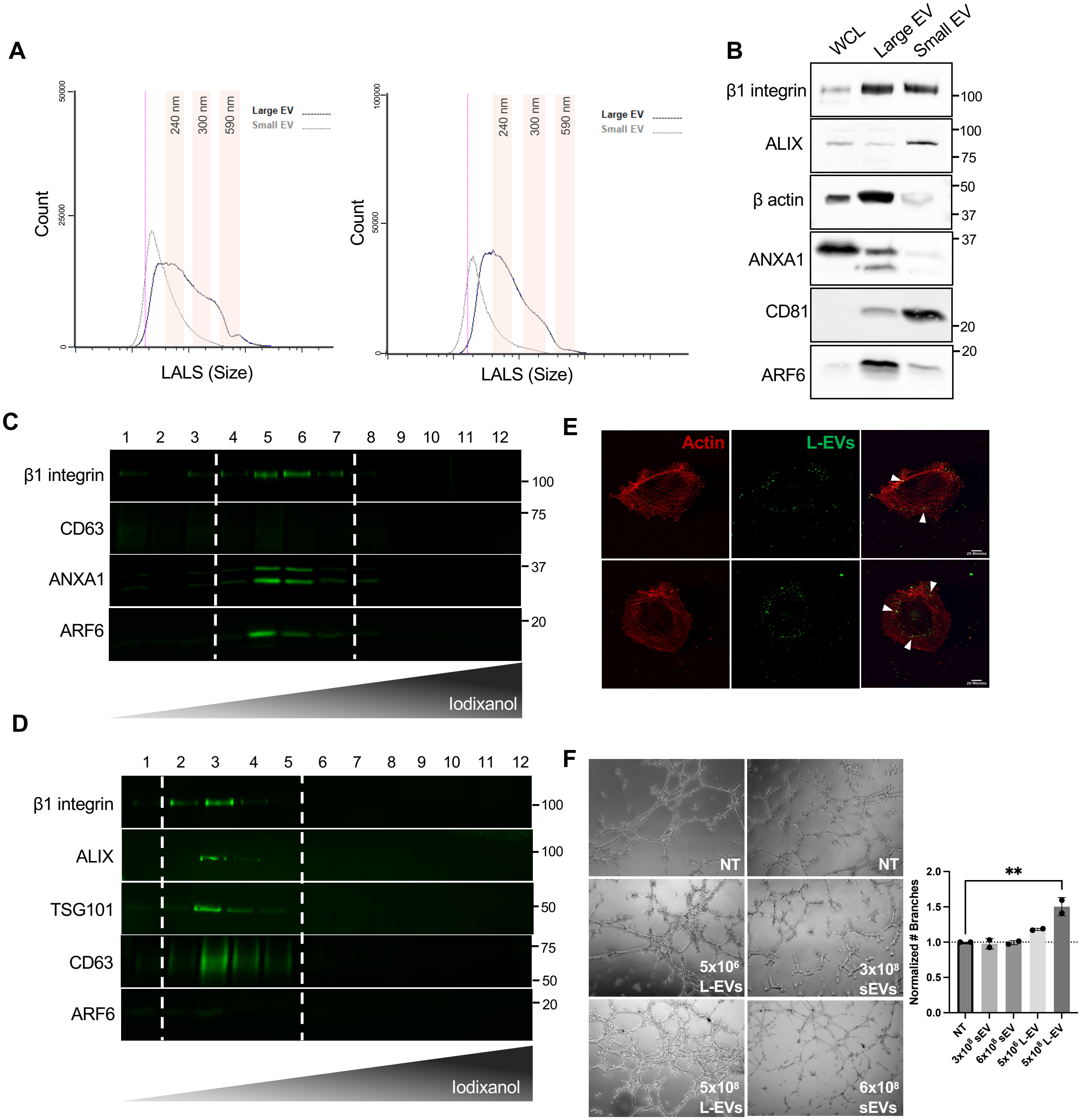

Richmond et al., Suppl. Fig. 1

A

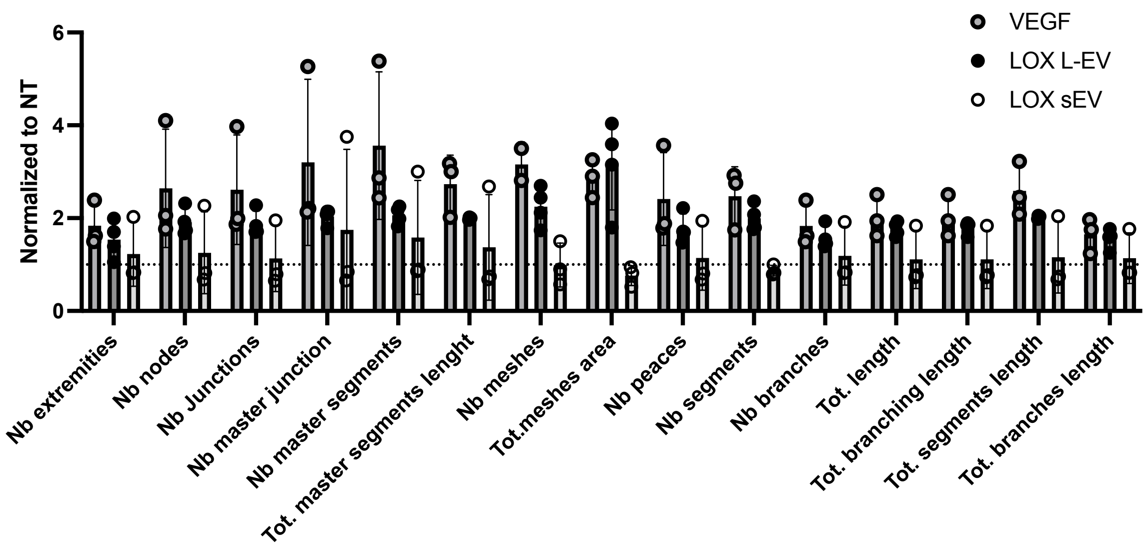

B

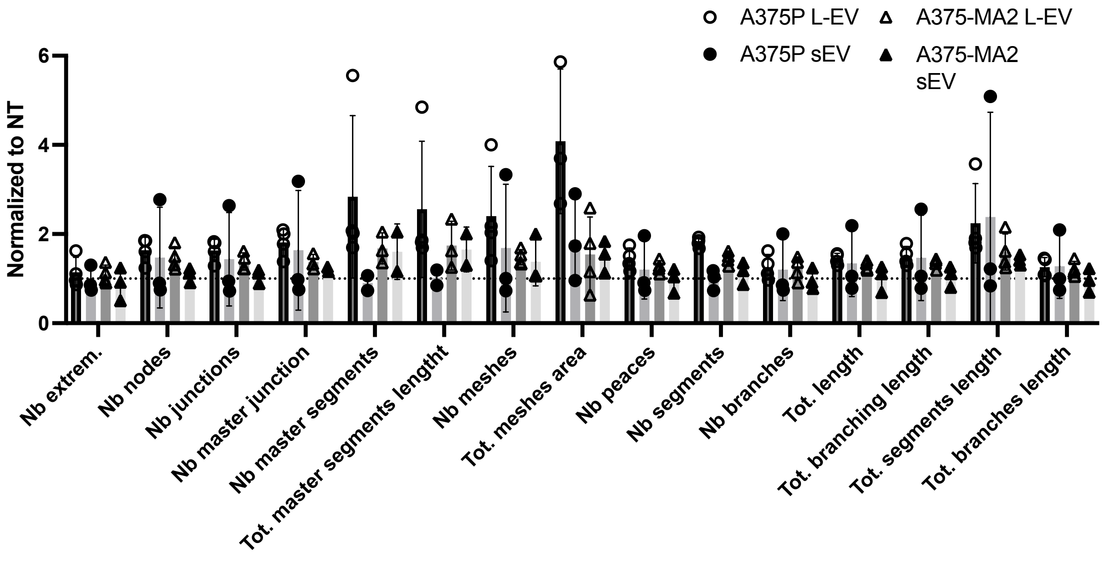

Richmond et al., Suppl. Fig. 2

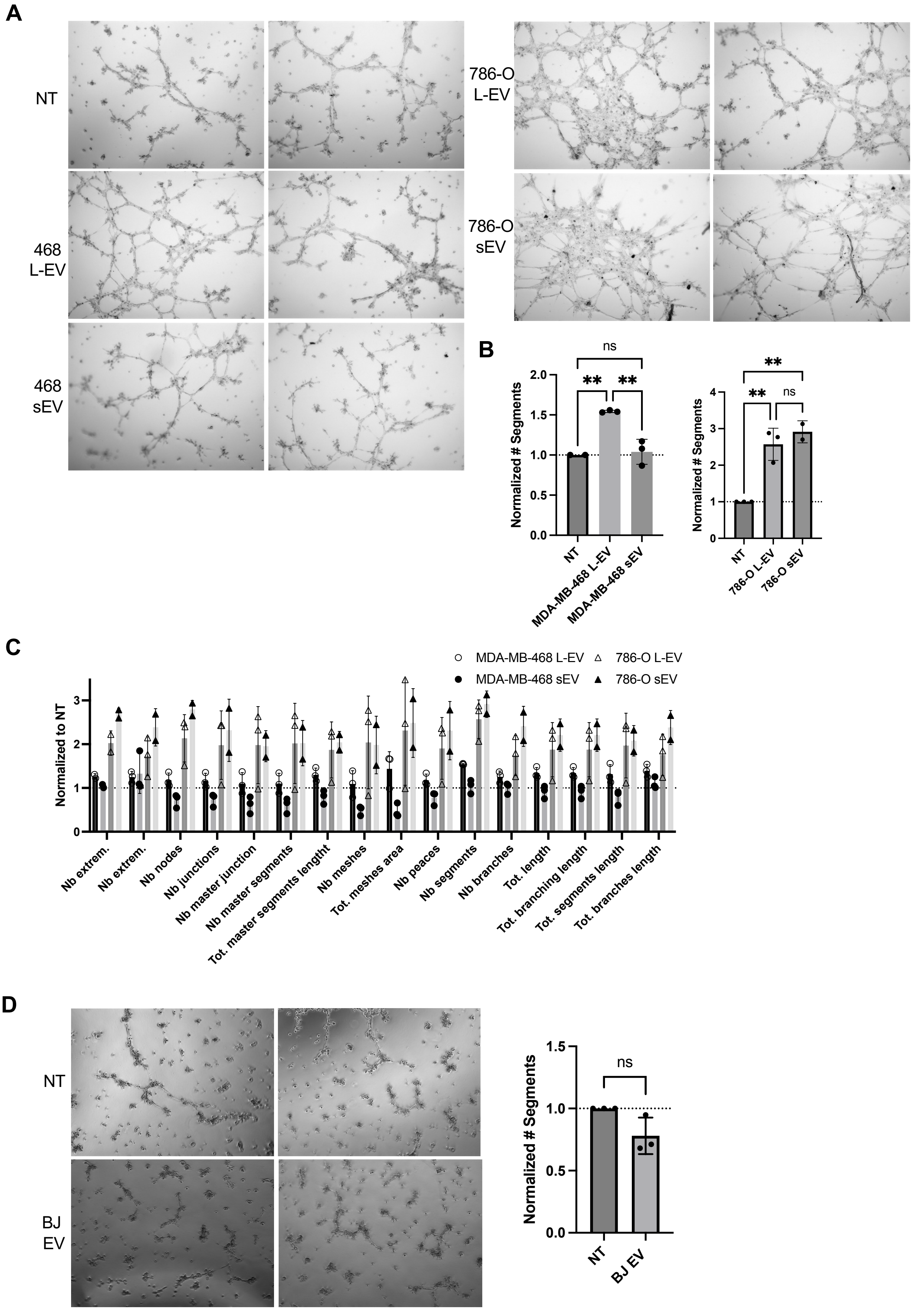

Richmond et al., Suppl. Fig. 3

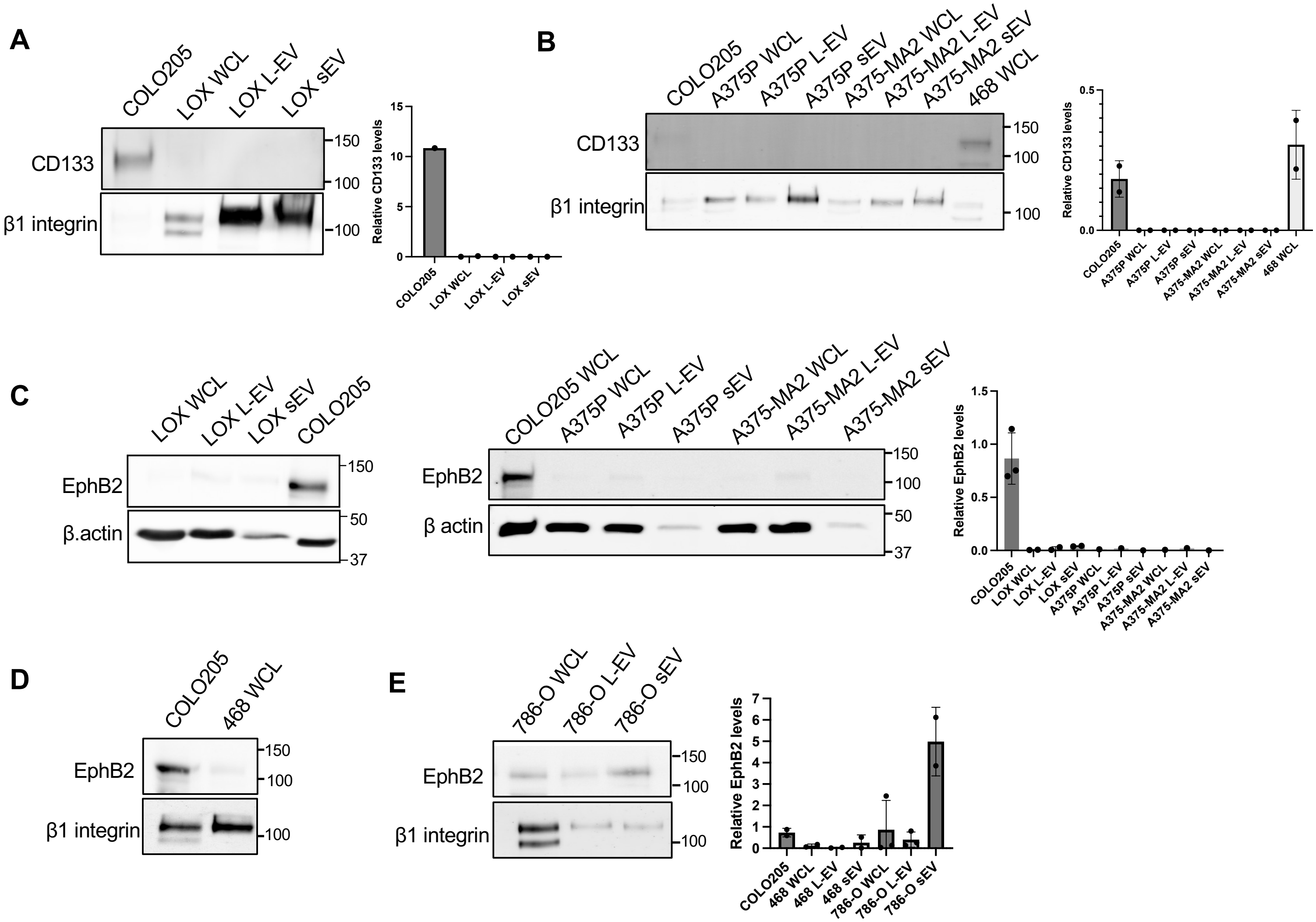

Richmond et al., Suppl. Fig. 4

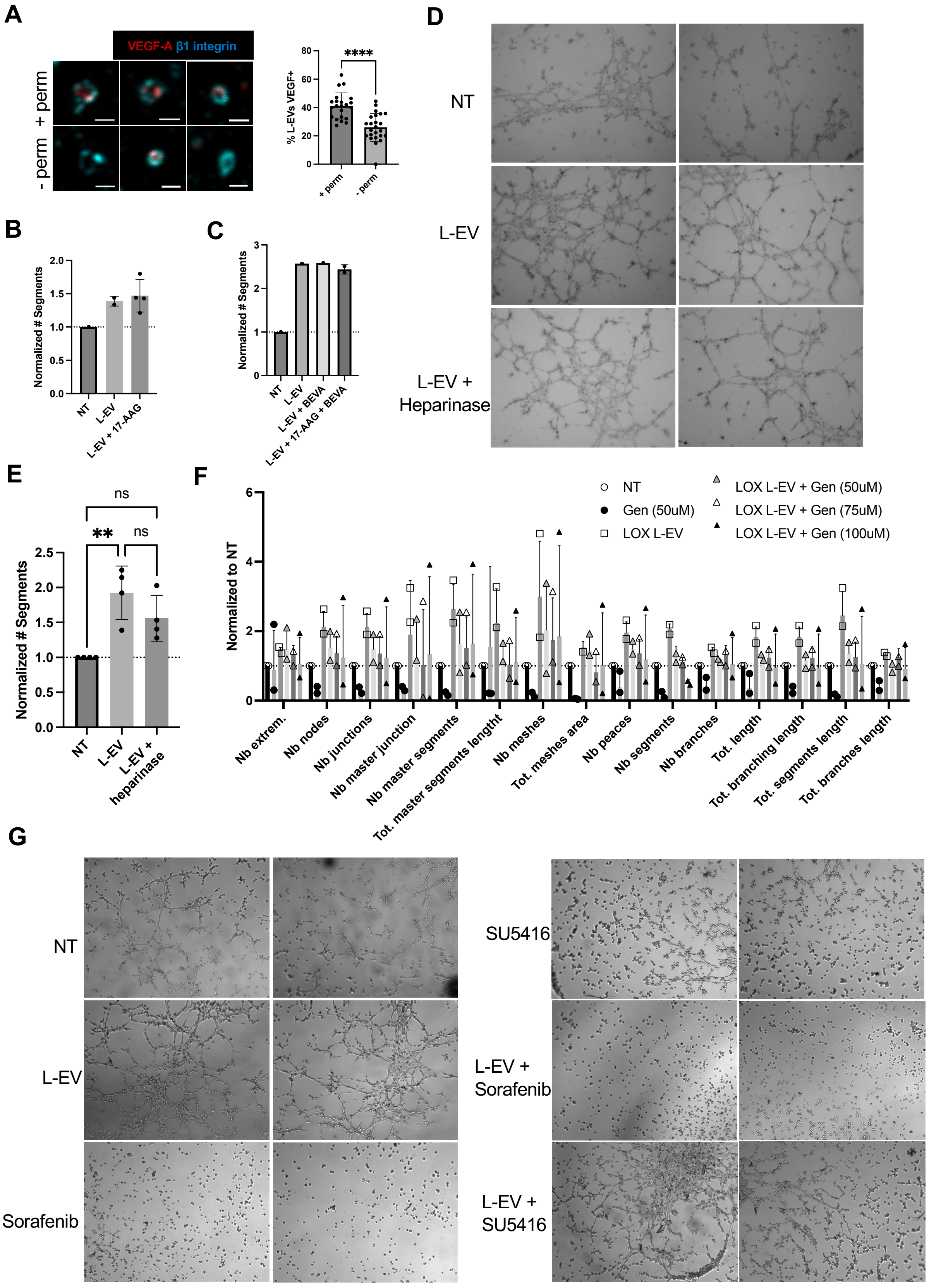

Richmond et al., Suppl. Fig. 5

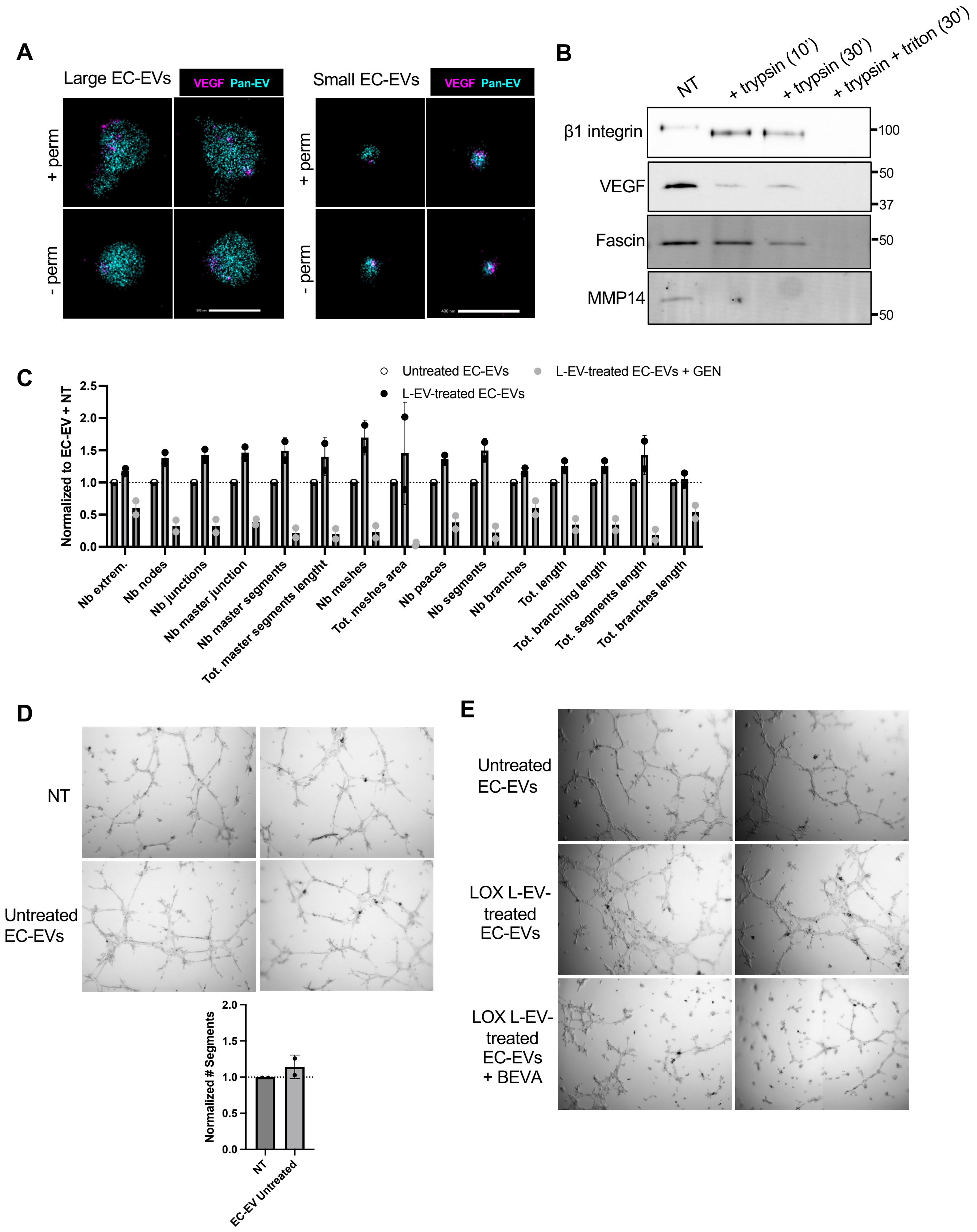

Richmond et al., Suppl. Fig. 6

A

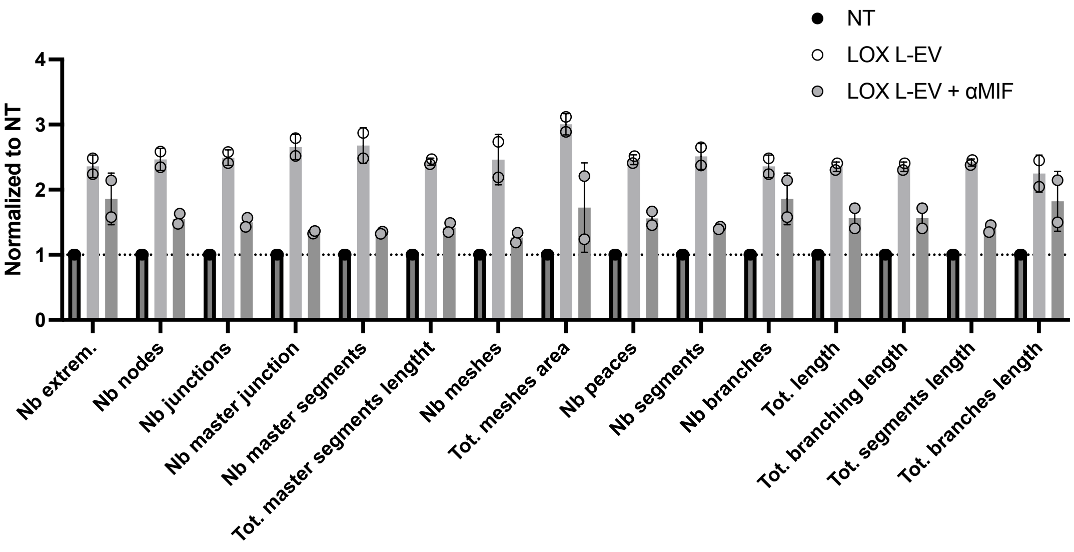

Richmond et al., Suppl. Fig. 7
